# Supplementary material for: THAP11F80L cobalamin disorder-associated mutation reveals normal and pathogenic THAP11 functions in gene expression and cell proliferation
Source: PLoS One. 2020 Jan 6;15(1):e0224646. doi: 10.1371/journal.pone.0224646 (PMC6944463; doi:10.1371/journal.pone.0224646)

All blots were visualized with the Odyssey R infra-red imaging system (LI-COR)

Figure 2A - upper panel

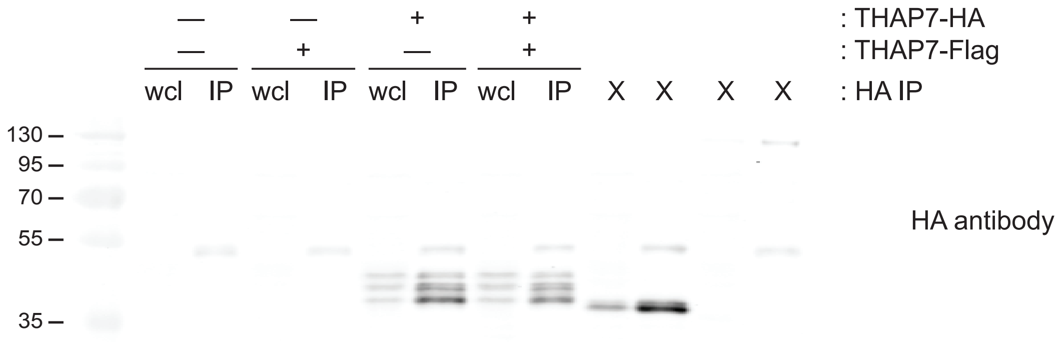

Figure 2A - lower panel

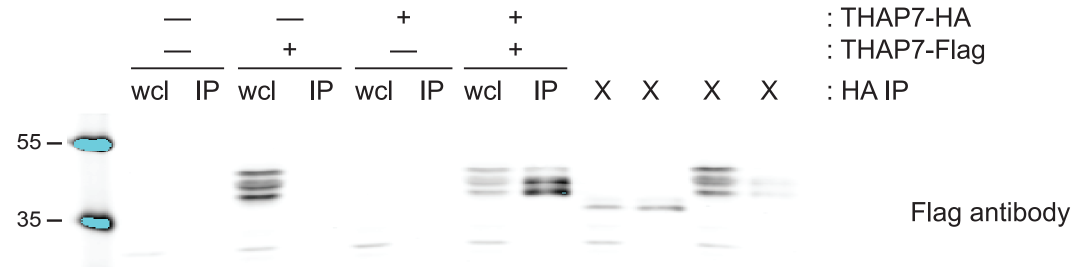

Figure 2B - upper panel

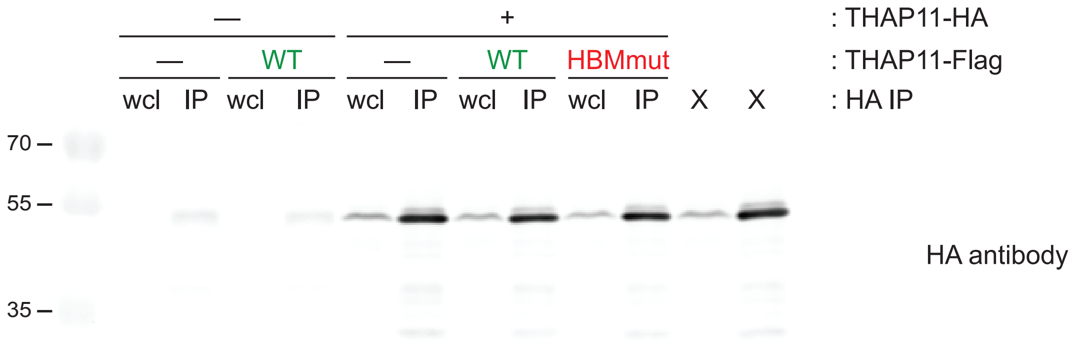

Figure 2B - lower panel

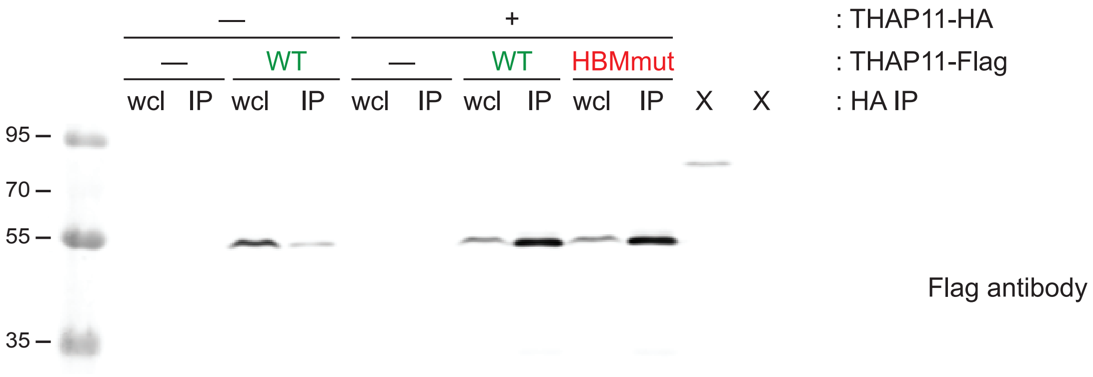

All blots were visualized with the Odyssey R infra-red imaging system (LI-COR)

Figure 2C - upper panel

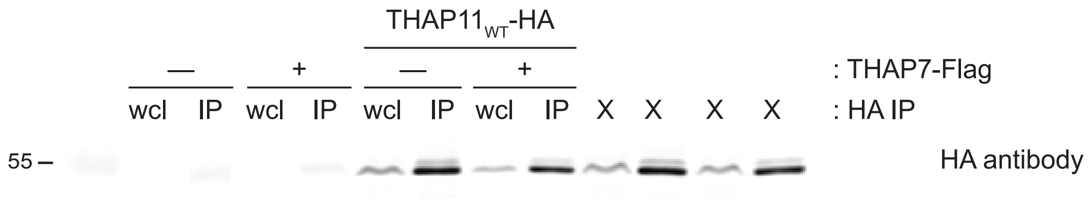

Figure 2C - lower panel

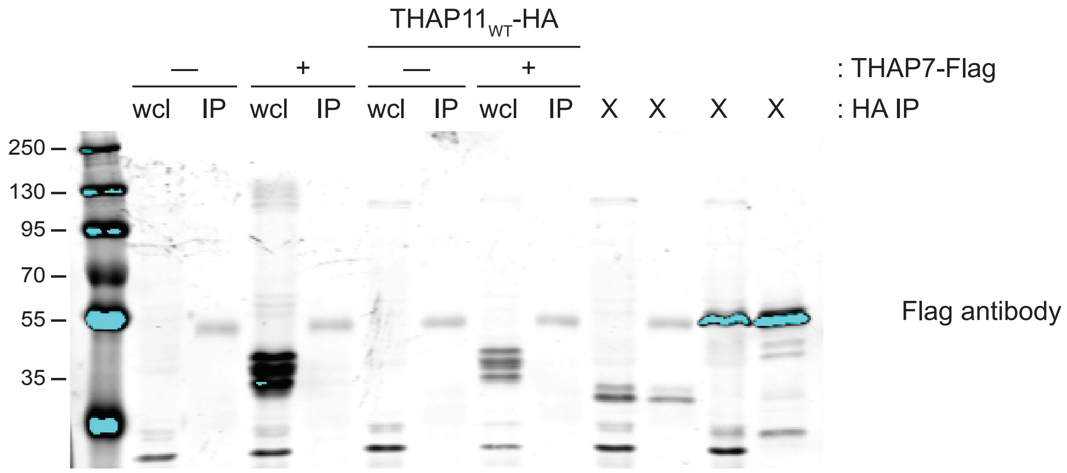

Figure 3A - upper panel

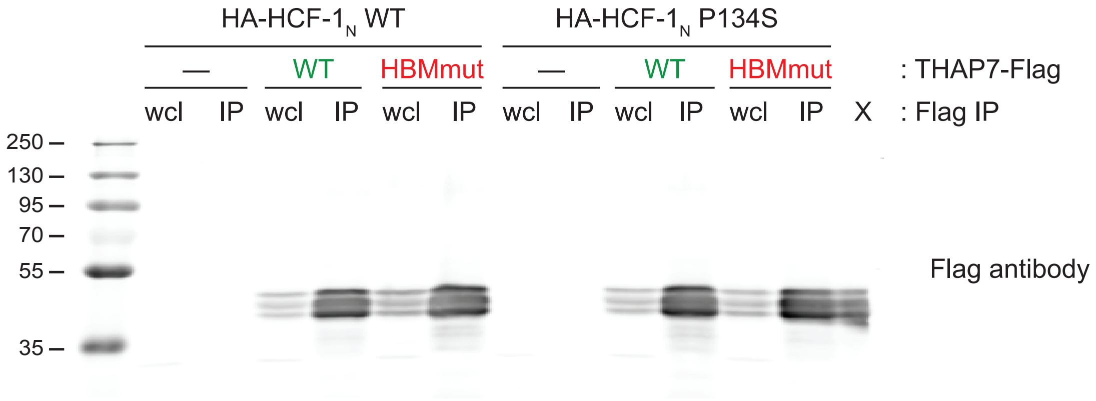

Figure 3A - lower panel

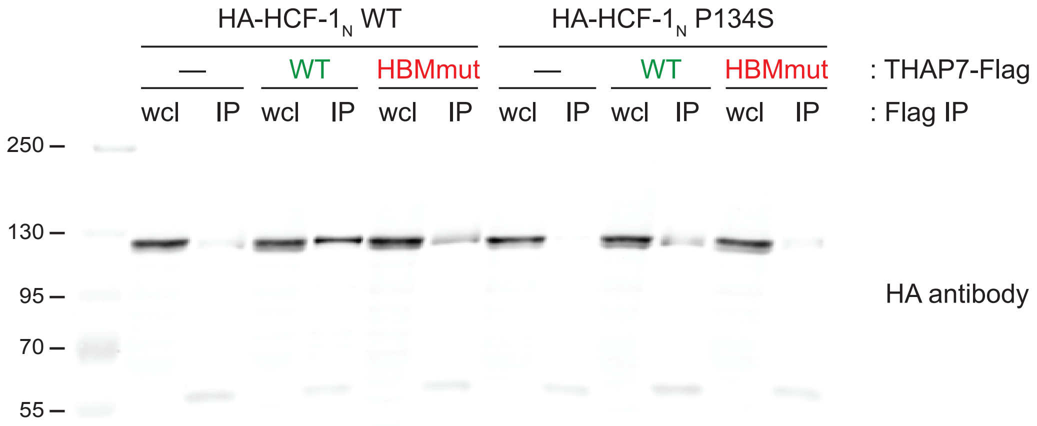

All blots were visualized with the Odyssey R infra-red imaging system (LI-COR)

Figure 3B - upper panel

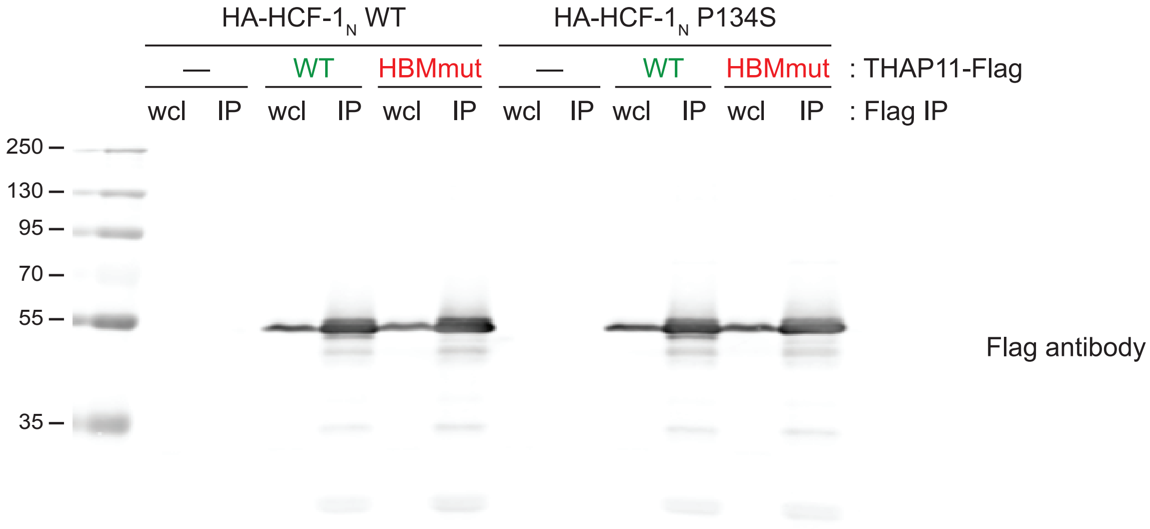

Figure 3B - lower panel

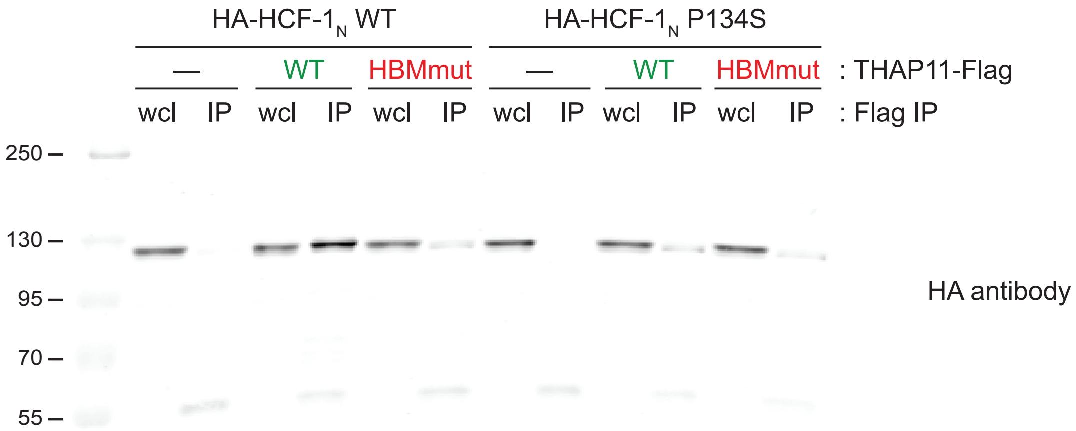

Figure 6C - upper panel

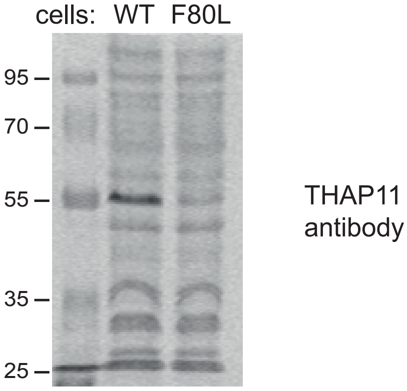

Figure 6C - lower panel

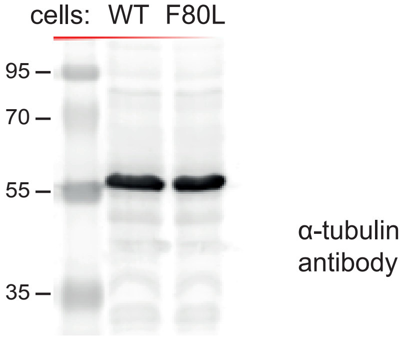

All blots were visualized with the Odyssey R infra-red imaging system (LI-COR)

S2A Fig

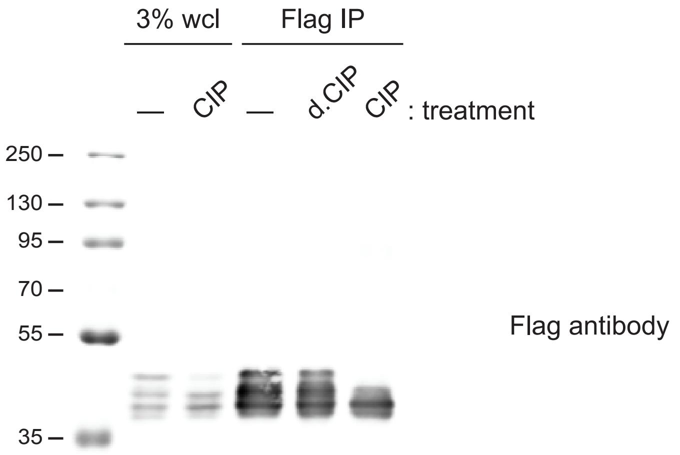

S2B Fig - upper and middle panels

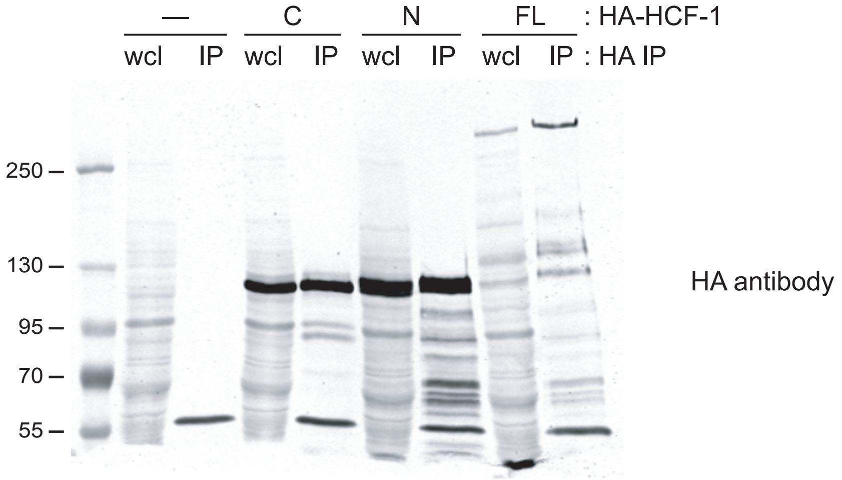

S2B Fig - lower panel

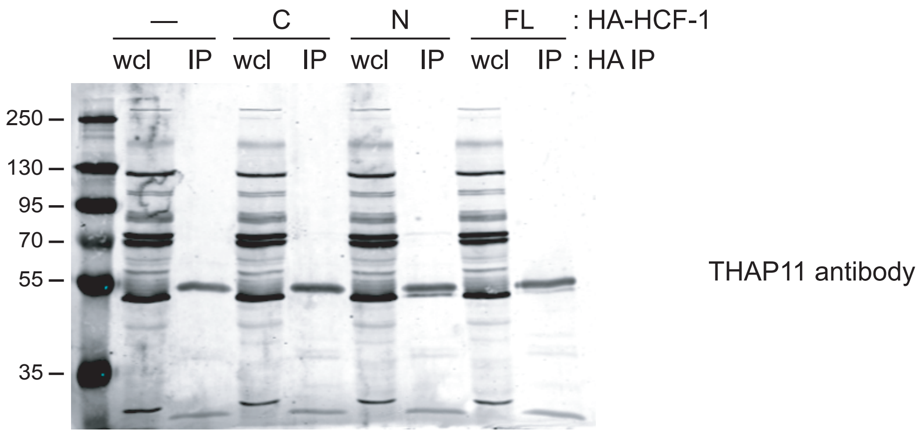

Supplement: S1 Raw Images — (PDF) [file pone.0224646.s001.pdf]
