# Supplementary material for: THAP11F80L cobalamin disorder-associated mutation reveals normal and pathogenic THAP11 functions in gene expression and cell proliferation
Source: PLoS One. 2020 Jan 6;15(1):e0224646. doi: 10.1371/journal.pone.0224646 (PMC6944463; doi:10.1371/journal.pone.0224646)
Supplement: S1 File — This file describes in detail the materials and methods used in the experiments reported in this article. (PDF) [file pone.0224646.s002.pdf]

Supporting information S1 File

Detailed materials and methods

Plasmids and site-directed mutagenesis

Plasmids

THAP7 and THAP11 WT open reading frames were purchased from GeneCopoeia™ and then cloned into the pcDNA™5/FRT/TO vector (Invitrogen V6520-20) with an T7-Flag tag or an HA tag at their C-termini. The HCF-1-encoding plasmids encode N-terminally HA and C-terminally c-myc-tagged HCF-1 versions (1). They are available from the Addgene plasmid repository: pCGN-HCF-1_{FL}, pCGN-HCF-$1N_{1011}$ (HA-HCF-1_N) and pCGN-HCF-$1C_{600}$ (HA-HCF-1_C).

Site-directed mutagenesis

THAP7 and THAP11 WT open reading frames were mutated to create HBM mutants. The EHSY (THAP7) and DHSY (THAP11) HBM sequences were mutated to EASA and DASA sequences, respectively. This corresponds to the H230A/Y232A mutation in THAP7, and the H244A/Y246A mutation in THAP11. For this, partially overlapping primer pairs were designed as recommended (2). A polymerase chain reaction (PCR) was performed with 100 ng of the template plasmid, 0.2 μM of each forward and reverse primer, 200 μM of dNTPs and 2.5 units of Pfu turbo Taq polymerase (Agilent 600250-52) in 1X Pfu buffer supplemented with Quick Solution (Agilent 200516-51). The mix was incubated in the thermocycler for the PCR, with an extension time of 1 minute per kilobase (kb) of template plasmid. At the end of the PCR reaction, the mix was incubated with 20 units of DpnI enzyme (New England Biolabs R0176S) to digest the (methylated) template plasmid. The mutagenesis mix was then used to transform competent bacteria under carbenicillin selection (100 μg/μL, Carl Roth 6344.1). DNA was

extracted from the bacteria and sequenced to assess the presence of the desired mutation in the plasmid.

Maintenance of cells in culture

Adherent cells were routinely maintained in DMEM medium (DMEM + 4.5g/L D-Glucose, L-Glutamine, Pyruvate; Gibco 419666) supplemented with 10% of heat-inactivated fetal calf serum (FCS, BioConcept 2-01F30) at 37°C and 5% CO_2 in a humidified atmosphere.

Cell proliferation assays

Cells were seeded (day 0) in wells of 6-well plates at 2.5 x 10^4 cells per well and incubated for 24 hours at 37°C to allow their attachment. For each condition, cells were plated in duplicate. Cells of two different wells per condition were then counted on day 1, and every day from day 4 to day 8.

For analysis, 20 μL of resuspended cells were mixed with 1 μL of solution 18 (Chemometec 910-3018); 10 μL of the mix was subsequently used for loading on an eight-chamber counting slide (Chemometec NC-Slide A8™ 942-0003) and automated cell counts and viability were determined with the automated cell analyser NucleoCounter® NC-250™ (Chemometec). Cell-count results were displayed as the ratio between the mean of live-cell number of the two replicates at the time point (N_t), and the cell number initially seeded on day 0 (N_0). Viability results were displayed as the percentage of live cells in the total cell number.

THAP7$_{HBM}$, THAP7$_{\Delta CC}$ and parental cell lines were tested in parallel in a first experiment, while THAP11$_{F80L}$, THAP7$_{null}$ and parental cell lines were tested in parallel in a second experiment.

Cell transfection for biochemistry analyses

For THAP-protein interactions analyses as described in Figs 2 and 3, cells were seeded in 10 cm dishes at 4 x 10^6 cells per dish 18 hours before transfection to allow their attachment. For the transfection, 6 μg of total DNA (3 μg of each plasmid in case of two plasmids) was resuspended into 250 μL of serum-free Opti-MEM medium (Gibco 31985). Separately, 10 μL of Lipofectamine® 2000 (Invitrogen 11668-019) was diluted into 250 μL of serum-free Opti-MEM medium. After 5 minutes incubation at room temperature, the Lipofectamine®-medium mix was added dropwise to the DNA-medium mix. After incubating for 20 minutes at room temperature, the DNA-Lipofectamine® mix was added dropwise onto the cells. The cells were then further incubated for 28 hours before harvesting for analysis.

Co-immunoprecipitation

Cells were on-plate lysed on the plate in 500 μL per plate of 0.5% NP40 lysis buffer (10 mM Tris pH 8.0, 150 mM NaCl, 5 mM MgCl2, 0.5% NP40, supplemented with one tablet of complete EDTA-free Protease Inhibitor Cocktail (Roche 04693132001) per 50 mL). From the lysate, 40 μL was transferred into a fresh tube for the whole-cell lysate (wcl) sample, mixed with 10 μL of 5X Laemmli buffer (250 mM Tris pH 6.8, 500 mM mercaptoethanol, 50% glycerol, 10% SDS, with bromophenol blue) and heated at 95°C for 5 minutes. The rest of the lysate was incubated overnight at 4°C with 30 μL of monoclonal anti-HA (Sigma A2095) or monoclonal

anti-Flag (Sigma A2220) agarose beads. After 4 washes of the beads with the 0.5% NP40 lysis buffer, the beads were resuspended in 20 μL of 5X Laemmli buffer, heated at 95°C for 5 minutes and the supernatant was used for immunoblotting (IP sample).

Immunoblotting

The samples were fractionnated by SDS-PAGE before being transferred onto a nitrocellulose membrane. For samples after co-immunoprecipitation, equal volumes of wcl and IP samples were loaded (5 μL, generating a wcl to IP ratio of 1/30). Membranes were blocked for 1 hour in 100% blocking buffer (LI-COR Bioscences 927-40000), and incubated overnight at 4°C with the primary antibody diluted 1/1000 in a mix of 50% PBS/0.5% Tween 20, and 50% blocking buffer. They were then washed with PBS/0.1% Tween 20 before being incubated with the appropriate secondary antibody for 1 hour at room temperature. Blots were visualized with the Odyssey R infra-red imaging system (LI-COR).

CRISPR/Cas9 mutagenesis

Design

Using the online CRISPR Design tool (http://tools.genome-engineering.org) (3), we selected for each mutation a suitable gRNA fulfilling the following criteria:

- being immediately followed by a 5'- NGG PAM sequence;
- being 20-nucleotides long if it already has a G at its 5', or 21-nucleotides long adding an extra G nucleotide at its 5' if it does not already have one;
- minimizing the off-target activities, meaning having a high score on the CRISPR Design tool output;

- close enough to the mutation site: i.e. the cutting site, which is 3 nucleotides upstream to the 5'- NGG PAM sequence, should not be farther than 11 nucleotides away from the mutation site;
- the PAM sequence is disrupted by the mutation or, alternatively, can be disrupted by a silent mutation.

The different gRNA sequences used in this work are listed below. Please note that the gRNA sequence can be designed on either the sense or the antisense strand and that both forward and reverse sequences were synthesized to create a double strand gRNA. For cloning purposes, we added a 5'- CACC sequence to the forward sequence and a 5'- AAAC sequence to of the reverse one. The resulting partially overlapping forward and reverse gRNA DNA sequences were synthesized by Microsynth. The extra nucleotides enabled for cloning of the annealed gRNA sequences into the pSpCas9(BB)-2A-GFP backbone plasmid (Addgene plasmid 48138) using the BsbI restriction enzyme. This gRNA-expression plasmid, designed by Ran and colleagues (3), encodes the invariant gRNA scaffold and cloning sites for insertion of the guide sequence, together with the Cas9 nuclease and a GFP cassette for selection purposes.

	gRNA					
**THAP7$_{null}$**	5′-	CACC	G	CGCCGCCGGCTGCTGCACAC		-3′
	3′-		C	GCGGCGGCCGACGACGTGTG	CAAA	-5′
**THAP7$_{HBM}$**	5′-	CACC	G	CCAGAATGAACACAGCTACC		-3′
	3′-		C	GGTCTTACTTGTGTCGATGG	CAAA	-5′
**THAP7$_{\Delta CC}$**	5′-	CACC		GCAGCGCCTTACTCTGGAAG		-3′
	3′-			CGTCGCGGAATGAGACCTTC	CAAA	-5′
**THAP11$_{null}$**	5′-	CACC	G	CAACAACTCGCACCGGGACA		-3′
	3′-		C	GTTGTTGAGCGTGGCCCTGT	CAAA	-5′
**THAP11$_{HBM}$**	5′-	CACC	G	CTGACGACAAGGAGTACGAA		-3′
	3′-		C	GACTGCTGTTCCTCATGCTT	CAAA	-5′
**THAP11$_{\Delta CC}$**	5′-	CACC	G	CTTGTCGTCAGGCACCACGG		-3′
	3′-		C	GAACAGCAGTCCGTGGTGCC	CAAA	-5′
**THAP11$_{F80L}$**	5′-	CACC		GCTCATTGACGCCGCGCAGC		-3′
	3′-			CGAGTAACTGCGGCGCGTCG	CAAA	-5′

List of the gRNA sequences used for CRISPR/Cas9 mutagenesis. Forward and reverse sequences designed to create the double-strand gRNA DNA that was subsequently cloned into the pSpCas9(BB)-2A-GFP backbone plasmid. Red, core guide sequence; Blue, 5'- G added if not already present in the 20-nucleotide guide sequence; Green, extra nucleotides added for BsbI-mediated cloning into pSpCas9(BB)-2A-GFP.

The mutagenic repair templates were designed as followed: from the mutation sites, 80 nucleotides upstream and downstream were taken to create an approximately 160-nucleotides long single-stranded DNA fragment (depending on the number of nucleotides mutated). The following table lists the different single-stranded DNA oligonucleotides (ssODNs) used, which were synthesized by Integrated DNA Technologies (IDT).

	ssODN
**THAP7$_{null}$**	5′- TGCCCGGAGAGCCGCTTGCGACTTAACTCCCGCCTCTTTCCCAGATG CCGCGTCACTGCTCCGCCGCCGGCTGCTGCACATGATAGACGCGCGAG ACGCGCAACCGCGGCATCTCCTTCCACAGGTCAGCGCGCGTGCGCCGC GGGCTCACGTGCGCATGCGCTAG
**THAP7$_{HBM}$**	5′- CTCTCGAACCACGGCCAGTCTCCCCTCAGCGTATATGCTGCGCCTG CCCCCACCCGCCGGAGCCTACATCCAGAATGAAGCCAGCGCCCAAGTG GGCAGCGCCTTACTCTGGAAGCGGCGAGCCGAGGCAGCCCTTGATGCC CTTGACAAGGCCCAGCGCCAGCTGCAGGC
**THAP7$_{\Delta CC}$**	5′- ATATGCTGCGCCTGCCCCCACCCGCCGGAGCCTACATCCAGAATGAA CACAGCTACCAGGTGGGCAGCGCCTTACTCTGGTAGTAGCGAGCCGAG GCAGCCCTTGATGCCCTTGACAAGGCCCAGCGCCAGCTGCAGGCCTGC AAGCGGCGGGAGCAGCGGCTGC
**THAP11$_{null}$**	5′- TGGGCCGGGCCGGGCCGCGCGGCGCAGCCATGCCTGGCTTTACGT GCTGCGTGCCAGGCTGCTACAACAACTCGCACCGGTAGTAAGCGCTGC ACTTCTACACGTTTCCAAAGGACGCTGAGTTGCGGCGCCTCTGGCTCA AGAACGTGTCGCGTGCCGGCGTCAG
**THAP11$_{HBM}$**	5′- GGCTGGAGGCTGCCGAGTGCCCTATGGGCCCCCAGTTGGTGGTGGT AGGGGAAGAGGGCTTCCCTGATACTGGCTCCGACGCTTCGGCCTCCTT GTCGTCAGGCACCACGGAGGAGGAGCTCCTGCGCAAGCTGAATGAGC AGCGGGACATCCTGGCTCTGATGGAAG
**THAP11$_{\Delta CC}$**	5′- AGTTGGTGGTGGTAGGGGAAGAGGGCTTCCCTGATACTGGCTCCGA CCATTCGTACTCCTTGTCGTCAGGCACCACGGAGTAGTAGCTCCTGCGC AAGCTGAATGAGCAGCGGGACATCCTGGCTCTGATGGAAGTGAAGATG AAAGAGATGAAAGGCAGCATT
**THAP11$_{F80L}$**	5′- CCCACCACAGGCCACCGTCTCTGCAGCGTTCACTTCCAGGGCGGC CGCAAGACCTACACGGTACGCGTCCCCACCATCTTGCCGCTGCGCGGC GTCAATGAGCGCAAAGTAGCGCGCAGACCCGCTGGGGCCGCGGCCGC CCCGCCGCAGGCAGCAGCAGC

List of ssODN repair templates used for CRISPR/Cas9 mutagenesis. The mutated residues are depicted in red in each sequence.

Mutagenesis

HEK-293 cells were co-transfected with the pSpCas9(gRNA)-2A-GFP plasmid encoding for the gRNA, the Cas9 nuclease and GFP together with the appropriate ssODN repair template. For this, low-passage HEK-293 cells were seeded in 6 cm plates at 0.4 x 10^6 cells per plate, and

incubated for 24 hours, after which cells were transfected as follows: 500 ng of pSpCas9(gRNA)-2A-GFP plasmid and 1 μL of the appropriate ssODN (10 μM) was diluted into 250 μL of serum-free Opti-MEM medium (Gibco 31985). Separately, 4 μL of Lipofectamine® 2000 (Invitrogen 11668-019) was diluted into 100 μL of serum-free Opti-MEM medium. After 5 minutes of incubation at room temperature, the Lipofectamine®-medium mix was added dropwise to the DNA-medium mix. After incubating for 20 minutes at room temperature, the DNA-Lipofectamine® mix was added dropwise onto the cells and the cells were further incubated for 3 to 4 days.

Cells were then sorted using the FACS Aria II instrument to select for the GFP-positive transfected cells. Upon exit from the cell sorter, cells were immediately separated at one cell per well in three 96-well plates, to allow for single-cell cloning. In addition, the remaining GFP-positive cells were collected and grown altogether for 2 to 3 days before being manually plated in three 96-well plates at an average density of 0.5 cell per well. Also, the same procedure was done in parallel for GFP-negative cells as a control.

Clones were isolated from several independent experiments as follows:

- Experiment A: $THAP7_{null}$ #29; $THAP7_{HBM}$ #34 and #76;
- Experiment B: $THAP7_{\Delta CC}$ #7, #27, #36 and #40; $THAP11_{F80L}$ #8;
- Experiment C: $THAP11_{HBM}$ #1;
- Experiment D: $THAP7_{null}$ #32 and #44.

Screening

Typically, 3 weeks after the cell sorting and the single-cell seeding, the media of some wells begins to turn yellow, indicating that the original cell in this well has survived and that the culture is reaching confluency. Each well was then split into two: two-thirds of its content was

used for DNA extraction and subsequent screening and the rest was maintained in culture. DNA was extracted as follows: cells were resuspended in 15 μL of basic solution (25 mM NaOH, 0.2 mM EDTA, pH 12.0) and incubated in a thermocycler, first for 15 minutes at 68°C, followed by 30 minutes at 98°C and cooling to 4°C. Finally, 12 μL of acid solution (40 mM Tris, pH 5.0) was added and the extracted DNA quantified.

The PCR was performed by mixing approximately 100 to 200 ng of the extracted DNA, with 0.4 μM of each forward and reverse primer, 200 μM of dNTPs and 0.4 units (0.2 μL) of high fidelity Q5 DNA polymerase (New England Biolabs M0491L) in 1X Q5 buffer (total volume of 25 μL). The PCR was done with appropriate annealing temperatures and extension times, which were optimized for each primer pair. The table below lists the primer pairs used for these PCRs with their corresponding PCR conditions: annealing temperature, extension time and other special condition if appropriate.

	PCR primers	PCR conditions
**$THAP7_{null}$**	fwd 5′- TCAAGAGAATCGGCTGGGAC<br>rvs 5′- CGAGGCGAGCAACAACTAGC	59.8 °C<br>6 seconds<br>2.5% DMSO
**$THAP7_{HBM}$**	fwd 5′- GCCTTAGCAGCCCCTTTTCAG<br>rvs 5′- TCAGTCTCAACCGCAGCCG	57 °C<br>6 seconds
**$THAP7_{\Delta CC}$**	fwd 5′- CGGAGCCTACATCCAGAATGAAC<br>rvs 5′- TCAGTCTCAACCGCAGCCG	58.6 °C<br>6 seconds
**$THAP11_{null}$**	fwd 5′- CGCAGCCATGCCTGGCTTTACG<br>rvs 5′- CCGCCCTGGAAGTGAACGCTGC	53 °C<br>7 seconds
**$THAP11_{HBM}$**	fwd 5′- CGGAGTTACAGGCTGCTACC<br>rvs 5′- CTTCTCACGCAGTTCTTCGC	55 °C<br>6 seconds
**$THAP11_{\Delta CC}$**	fwd 5′- GCGTCGGAGTTACAGGCTG<br>rvs 5′- CCTTCTCACGCAGTTCTTCG	53.2 °C<br>6 seconds
**$THAP11_{F80L}$**	fwd 5′- CGTTTCCAAAGGACGCTGAGTTGC<br>rvs 5′- GCTGTTGCTGCTGCTGCCTGCG	70.6 °C<br>6 seconds

List of the PCR primer pairs used to screen the cell clones obtained after CRISPR/Cas9 mutagenesis, and their specific working conditions: annealing temperature (first lane), extension time (second line) and DMSO concentration if applicable (third lane). fwd, forward; rvs, reverse.

Following the PCR, half of the reaction mix was used for digestion with 4 units of appropriate restriction enzyme in the recommended 1X reaction buffer. The table below lists the different enzymes used for each reaction, together with the incubation conditions (buffer, temperature and duration). All enzymes and buffers were purchased from New England Bioloabs (NEB).

	Enzyme	Buffer	Temperature	Duration
**$THAP7_{null}$**	NlaIII	CutSmart buffer	37°C	O/N
**$THAP7_{HBM}$**	AluI	CutSmart buffer	37°C	1 hour
**$THAP7_{\Delta CC}$**	AciI	CutSmart buffer	37°C	1 hour
**$THAP11_{null}$**	AgeI	CutSmart buffer	37°C	1 hour
**$THAP11_{HBM}$**	CviQI	NEBuffer 3.1	25°C	1 hour
**$THAP11_{\Delta CC}$**	SacI	CutSmart buffer	37°C	1 hour
**$THAP11_{F80L}$**	FauI	CutSmart buffer	55°C	1 hour

Restriction enzymes used to screen the cell clones obtained after CRISPR/Cas9 mutagenesis, and their specific working conditions. O/N, overnight.

Following digestion, the PCR and corresponding digestion products were loaded side by side on a 2% agarose gel. When a clone displayed the expected digestion pattern for the mutation, the undigested PCR products were excised from the gel and incubated overnight in 40 μL of 1 M Tris, pH 6.8. The following day, the DNA released in the Tris buffer was sequenced using either the forward or reverse primer to confirm the presence of the mutation.

High-throughput RNA sequencing (RNA-seq)

For gene expression analysis of the mutant cells, 1.5 x 10^5 cells per well were seeded in 6-well plates and incubated at 37°C for 72 hours before analysis. Total RNA was extracted using the Qiagen RNeasy kit (Qiagen 74104) according to the manufacturer protocol. The RNA was

eventually eluted in water, quantified and subjected to quality control with a fragment analyser (Fragment Analyzer Automated CE System, Advanced Analytical Technologies). Ribosomal RNA was removed using the Ribo-zero rRNA removal kit (Illumina MRZH11124), libraries prepared using the truSEQ stranded RNA LT kit (Illumina) and 125-nucleotide single-read high-throughput sequencing was performed with 6 samples per line (multiplexing).

Chromatin immunoprecipitation followed by high-throughput sequencing (ChiP-seq)

Chromatin preparation

Cells were expanded at 37°C and approximately 1.5 x 10^8 cells were used per IP. Cells were crosslinked in situ with 1% formaldehyde (Sigma F1635-500ml) during precisely 8 minutes, then Glycine (Axonlab A1067.5000) was added to a final concentration of 0.125 M of to terminate the crosslinking reaction. Cells were washed twice with cold 1X PBS and lysed for 10 minutes on ice in 0.5% NP40 lysis buffer (5 mM PIPES pH 8.0, 85 mM KCl, 0.5% NP40, supplemented with one tablet of complete EDTA-free Protease Inhibitor Cocktail (Roche 04693132001) per 50 mL; 950 μL per 10 millions of cells). The nuclei were recovered by high-speed centrifugation (5 minutes at 3200 x g, at 4 C), resuspended in nuclei lysis buffer (NLB, 50 mM Tris-HCl pH 8.1, 10 mM EDTA pH 8.0, 1% SDS, supplemented with one tablet of complete EDTA-free Protease Inhibitor Cocktail) and incubated for 20 minutes at 4°C.

Chromatin was sonicated using a Bioruptor Pico (Diagenode) to obtain fragments about 200 bp long. Sonicated chromatin was clarified by centrifugation and subsequently diluted 1:2 in 2X IP buffer (2X IP buffer: 33.4 mM Tris pH 8.1, 167 mM NaCl, 167 mM LiCl, 2.4 mM EDTA pH 8.0, 2.2% Triton X-100, 0.02% SDS, supplemented with complete EDTA-free

Protease Inhibitor Cocktail) before being snap frozen in liquid nitrogen. A 50 μL aliquot was kept to analyze the chromatin quality and concentration. The frozen sonicated chromatin was stored at - 80°C.

Chromatin immunoprecipitation

The day prior to the ChIP, protein G agarose beads (Roche 1243233) were washed with NLB : 2X IP buffer (a mix of equal amounts of NLB and 2X IP buffer) and further incubated overnight under rotation at 4°C) in NLB : 2X IP buffer supplemented with 100 μg/mL of bovine serum albumin (BSA, Sigma A8022-100).

The frozen sonicated chromatin was thawed. A 60 μL aliquot was kept for the input sample, while, for each IP, 9 μg of chromatin (in a total volume of 1200 μL of NLB : 2X IP buffer) was incubated overnight with rotation at 4°C with 2 μg of anti-THAP11 antibody. Samples were further incubated with rotation at room temperature with 60 μL of the above washed and BSA-blocked protein G agarose beads. Immunoprecipitated samples were washed twice with IPWB1 buffer (IP wash buffer 1: 20 mM Tris pH 8.1, 50 mM NaCl, 2 mM EDTA pH 8.0, 1% Triton X-100 and 0.1% SDS). Each IP was performed 5 times in parallel as described and subsequently pooled to form a single IP sample at this step, after the washes with IPWB1 buffer. Pooled IP samples were then washed once with IPWB2 buffer (IP wash buffer 2: 10 mM Tris pH 8.1, 250 mM LiCl, 1 mM EDTA pH 8.0, 1% NP40 and 1% Na-deoxycholate) and finally twice with TE buffer (10 mM Tris pH 8.1, 1 mM EDTA). Two elutions with 125 μL each of IPEB buffer (IPEB elution buffer: 100 mM NaHCO3, 1% SDS) were done sequentially (5 minutes at 37°C with agitation) and pooled.

The input sample was thawed and supplemented with 190 μL of IPEB buffer. Eluates (IP) and input samples were decrosslinked by overnight incubation at 65°C in the presence of 20 μg/mL of RNAse A (DNAse-free RNAse A, Roche 1119915) and 300 mM of NaCl. The

samples were further incubated during 90 minutes at 45°C with 350 μg/mL of proteinase K (Promega V3021). Samples were finally purified using the Nucleospin Gel and PCR clean-up kit (Macherey-Nagel 740609) using the NTB buffer for SDS-containing samples. Samples were eluted with 50 μL of the pre-warmed (72°C) buffer NE (from the kit), and subsequently re-eluted using the previous eluate. DNA yield was quantified using a Qubit spectrophotometer.

Library preparation and sequencing

For each IP sample, two separate libraries were prepared and sequenced, using the same immunoprecipitated-DNA material. For this, 5 ng of purified DNA was used to prepare paired-end sequencing libraries using the MicroPlex Library Preparation kit (Diagenode C05010014) following the manufacturer instructions. Here, 8 PCR cycles were done for DNA amplification and the DNA fragments were not size selected. Then, libraries were purified using AMPure® XP magnetic beads (Diagenode) and subjected to 100-nucleotide paired-end high-throughput sequencing (Illumina, HiSeq 2100) with 3 samples per lane (multiplexing).

Antibodies

The antibodies used were: anti-HA (rat, Roche 10768600), anti-Flag (rabbit, Cell Signaling 2368S), anti- HCF-1 (rabbit, H12 (4)), anti-OGT (rabbit, Santa Cruz Biotechnology sc-32921), anti-tubulin (mouse, Sigma T0198) and anti-THAP11 (sheep, R & D Systems AF5727).

Bioinformatics

Analysis of THAP proteins

The presence of an HBM sequence in the THAP proteins was assessed using Protein Pattern Find (5), providing a fasta file with the THAP protein sequences as a query, and looking at

consensus HBM sequences ($^{B}/_{Z}$HxY, where B and Z are aspartate/asparagine or glutamate/glutamine, respectively, and x denotes any amino-acid) using the following search pattern: “[DNEQ]H.Y”.

The presence of a coiled-coil domain in the THAP proteins was assessed with two separate tools: COILS (6) and PairCoil2 (7). Fasta sequences of the THAP proteins were used as queries, with default parameters, except for the p-score cut-off which was set to 0.05. Both tools gave identical results in terms of presence or absence of a coiled-coil domain, even though precise coiled-coil domain boundaries may slightly differ.

The evolutionary tree (Fig 1) was done using the Mobyle portal (8) to perform multiple alignment (Muscle alignment), by providing an input fasta file containing the sequences to be aligned (the 90 N-terminal amino-acids). The alignment was refined using the “protein boostrap distance phylogeny (alignment)” option (random number seed = 3; random number seed for multiple dataset = 7). The output of the alignment was subsequently uploaded on the iTOL (interactive Tree Of Life) online tool (9,10) for visualization.

The alignment of human THAP proteins depicted in S5 Fig was done using Jalview, after embedded multiple alignment (Muscle alignment).

RNA-seq analysis

Single reads fro the high-throughput RNA sequencing were mapped onto the Hg19 human genome annotation using STAR (Spliced Transcripts Alignment to a Reference, (11)) and read counts and normalized RPKM (Reads Per Kilobase of transcript per Million mapped reads) were calculated using RSEM (12,13). Only protein-coding genes were considered; genes with a corresponding RPKM value below 1.2 in all the samples were considered as not expressed and removed from further analyses. Differential analyses were performed with DESeq2 (14)

(fold-change cut-off = 0.5, adjusted p-value = 0.05). Resulting gene sets were submitted to Gene Ontology (GO) enrichment analysis (15,16).

ChIP-seq analysis

Paired-end reads from the ChIP-seq analysis were mapped onto the Hg19 human genome annotation using STAR (Spliced Transcripts Alignment to a Reference) (11). Each fragment end was mapped individually allowing a maximum of 10 multiple genomic matches. Only the equivalent multiple matches (in terms of match length and mismatch numbers) were kept. The sequencing pairs were then reassembled with in-house scripts.

Duplicate sequencing data were treated as follows. For each cell line, the sum of the peaks found in the duplicates were considered, with peaks present in both replicates only counted once. If needed to choose between the peak data coming from one or the other replicate, sample (a) was used. When quantifying the peaks, the mean between the replicates was considered. Finally, a single replicate per sample is shown (replicate (a) for each sample) for vizualisation with the UCSC Genome Browser.

Peaks were detected using the Model-based Analysis of ChIP-Seq (MACS2) tool (17) (format = BEDPE, q-value cut-off = 10^{-3}, duplicates allowed = 3, Broad region calling = off) and tested by the “Origami method” (V. Praz, see (18)). Only MACS-identified peaks intersecting with the Origami enriched bins were kept. Peaks were further classified into 3 categories: (i) peaks present in both the $THAP11_{WT}$ parental and the $THAP11_{F80L}$ mutant samples called “common”, (ii) peaks missing in the $THAP11_{F80L}$ sample called “F80L-absent”, and (iii) peaks present exclusively in the $THAP11_{F80L}$ sample called “F80L-only”. A peak was said to be close to a TSS if at least one nucleotide of its underlying DNA sequence was located within +/- 250 base pairs of a RNA polymerase II transcription start site.

Visualization of data using Venn diagrams was done using Meta-Chart.

Peak scores were calculated as follows: (i) peak fragments of the IP sample were summed on the whole peak region, and normalized to total fragments in the sample and peak width (bringing all peaks width at an arbitrary 1 kb size); (ii) the same procedure was done for the input sample; (iii) for each sample, the peak score was calculated as the log2 of the normalized IP counts (done in (i)) minus the input normalized counts (done in (ii)). For calculation purposes, one pseudocount was added to each of the IP and input normalized counts.

Sequence comparison with known motifs was done on regions expanding 500 bp on each side of the peak middle using CentriMo (19).

The motifs under the THAP11 peaks were further analyzed to identify subtle differences in the consensus sequences. First, the peaks were redefined by taking the peak summits (meaning, the highest position of the peaks) as a reference (instead of the peak centers) and extending the positions 250 bp on each side. Second, the SBS2 motif from Hocomoco (reference ZN143 HUMAN.H11MO.0.A, here 22-bp long) was screened over the whole genome using the PWMTools web interface (20) to extract all the genomic positions containing such motif, as well as the corresponding 22-bp DNA sequence. Third, sequences intersecting between the list of peaks and the genomic screen described (the latter having produced a list of more than 9 million of sequences) were extracted. If more than one motif was identified for a given peak, only the one closest to the peak center was considered. Fourth, a consensus sequence was generated using the list of pre-aligned 22-bp long sequences and a logo was made. The number of THAP11-associated motifs per peak was defined by counting, for each peak, the number of motifs in a region extending 1000 bp on each side of the peak maximum.

Peaks were visualized with the UCSC genome browser (21) using uploaded wiggle files, all tracks being set with the same vertical viewing range (which varies depending on the various visualizations).

1. Wilson AC, Freiman RN, Goto H, Nishimoto T, Herr W. VP16 targets an amino-terminal domain of HCF involved in cell cycle progression. Mol Cell Biol. 1997 Oct;17(10):6139–46.

2. Zheng L, Baumann U, Reymond JL. An efficient one-step site-directed and site-saturation mutagenesis protocol. Nucleic Acids Res. 2004;32(14).

3. Ran FA, Hsu PD, Wright J, Agarwala V, Scott DA, Zhang F. Genome engineering using the CRISPR-Cas9 system. Nat Protoc. 2013;8(11):2281–308.

4. Wilson AC, LaMarco K, Peterson MG, Herr W. The VP16 accessory protein HCF is a family of polypeptides processed from a large precursor protein. Cell. 1993;74(1):115–25.

5. Stothard P. The sequence manipulation suite: JavaScript programs for analyzing and formatting protein and DNA sequences. Biotechniques. 2000 Jun;28(6):1102, 1104.

6. Lupas A, Van Dyke M, Stock J. Predicting coiled coils from protein sequences. Science. 1991 May 24;252(5009):1162–4.

7. McDonnell A V, Jiang T, Keating AE, Berger B. Paircoil2: improved prediction of coiled coils from sequence. Bioinformatics. 2006 Feb 1;22(3):356–8.

8. Edgar RC. MUSCLE: multiple sequence alignment with high accuracy and high throughput. Nucleic Acids Res. 2004;32(5):1792–7.

9. Letunic I, Bork P. Interactive Tree Of Life (iTOL): an online tool for phylogenetic tree display and annotation. Bioinformatics. 2007 Jan 1;23(1):127–8.

10. Letunic I, Bork P. Interactive Tree Of Life v2: online annotation and display of phylogenetic trees made easy. Nucleic Acids Res. 2011 Jul;39(Web Server issue):W475-8.

11. Dobin A, Davis CA, Schlesinger F, Drenkow J, Zaleski C, Jha S, et al. STAR: ultrafast universal RNA-seq aligner. Bioinformatics. 2013 Jan 1;29(1):15–21.

12. Li B, Dewey CN. RSEM: accurate transcript quantification from RNA-Seq data with or without a reference genome. BMC Bioinformatics. 2011 Aug 4;12:323.

13. Li B, Ruotti V, Stewart RM, Thomson JA, Dewey CN. RNA-Seq gene expression estimation with read mapping uncertainty. Bioinformatics. 2010 Feb 15;26(4):493–500.

14. Love MI, Huber W, Anders S. Moderated estimation of fold change and dispersion for RNA-seq data with DESeq2. Genome Biol. 2014 Dec 5;15(12):550.

15. The Gene Ontology Consortium. Expansion of the Gene Ontology knowledgebase and resources. Nucleic Acids Res. 2017;45(D1):D331–8.

16. Ashburner M, Ball CA, Blake JA, Botstein D, Butler H, Cherry JM, et al. Gene ontology: tool for the unification of biology. The Gene Ontology Consortium. Nat Genet. 2000 May;25(1):25–9.

17. Zhang Y, Liu T, Meyer CA, Eeckhoute J, Johnson DS, Bernstein BE, et al. Model-based analysis of ChIP-Seq (MACS). Genome Biol. 2008;9(9):R137.

18. Renaud M, Praz V, Vieu E, Florens L, Washburn MP, L'Hôte P, et al. Gene duplication and neofunctionalization: POLR3G and POLR3GL. Genome Res. 2014 Jan;24(1):37–51.

19. Bailey TL, Machanick P. Inferring direct DNA binding from ChIP-seq. Nucleic Acids Res. 2012;40(17):e128.

20. Ambrosini G, Groux R, Bucher P. PWMScan: a fast tool for scanning entire genomes with a position-specific weight matrix. Bioinformatics. 2018;34(14):2483–4.

21. Kent WJ, Sugnet CW, Furey TS, Roskin KM, Pringle TH, Zahler AM, et al. The Human Genome Browser at UCSC. Genome Res. 2002 May 16;12(6):996–1006.
