# Supplementary material for: THAP11F80L cobalamin disorder-associated mutation reveals normal and pathogenic THAP11 functions in gene expression and cell proliferation
Source: PLoS One. 2020 Jan 6;15(1):e0224646. doi: 10.1371/journal.pone.0224646 (PMC6944463; doi:10.1371/journal.pone.0224646)
Supplement: S3 Table — Features of the 10 THAP11 peaks in a 3.3 Mb region of Chromosome 1 including the MMACHC gene. Relative to Fig 7C. (PDF) [file pone.0224646.s012.pdf]

			ChIP-seq data			RNA-seq data									
			peak scores (1)			S gene (2)					A-S gene (2)				
Peak number in Fig 7	unique ID	peak category	$THAP11_{WT}$	$THAP11_{F80L}$	fold change	name	distance to peak (3)	RNA-seq log2(RPKM) $THAP11_{WT}$ (4)	RNA-seq log2(RPKM) $THAP11_{F80L}$ (4)	log2 fold change $THAP11_{WT}$ vs $THAP11_{F80L}$	name	distance to peak (3)	RNA-seq log2(RPKM) $THAP11_{WT}$ (4)	RNA-seq log2(RPKM) $THAP11_{F80L}$ (4)	log2 fold change $THAP11_{WT}$ vs $THAP11_{F80L}$
1	2061	common	8,72	9,03	-0,31	*ZNF691*	107	3,39	3,41	0,00	*RP11-342M1*	-80	0,44	1,02	0,51
2	1181	common	10,67	10,10	0,57	*IPO13*	196	4,04	4,06	-0,01	*RP11-7O11,3*	-192	ND	ND	ND
3	2356	common	9,51	10,28	-0,77	*DPH2*	277	4,76	4,81	0,03					
4	1430	common	9,48	9,18	0,30	*ATP6V0B*	-262	6,69	6,61	-0,13					
5	769	common	9,44	8,02	1,42						*ERI3*	-220	6,27	6,08	-0,21
6	655	common	9,95	8,18	1,76	*RNF220*	53	6,04	6,17	0,08					
*	312	F80L absent	8,56	4,67	3,89	*BTBD19*	NA	ND	ND	ND					
7	45	F80L absent	8,60	2,50	6,10	*TOE1*	NA	0,19	0,19	0,19	*MUTYH*	113	3,40	1,79	-1,86
8	4	F80L absent	9,14	1,10	8,04	*MMACHC*	-176	3,32	1,22	-2,17	*CCDC163P*	-150	ND	ND	ND
9	573	common	11,29	9,19	2,09	*TMEM69*	73	4,77	5,03	0,15	*GPBP1L1*	NA	6,11	6,51	0,41

1 See Materials and Methods for the description of score calculation
2 S gene indicate the closest gene (+/-250 bp) that is a sense transcript ; A-S gene indicate anti sense transcripts.
3 distance between the gene transcription start site and the pek maximum
4 Mean between the two biological replicates
GENE gene which transcription start site is within a region extending 250 bp on each side of the peak boundaries
GENE gene which transcription start site is outside the aforementionned region
NA non-applicable, meaning no such gene relative to the peak
ND non-determined, meaning gene not expressed in our dataset
