# Supplementary material for: THAP11F80L cobalamin disorder-associated mutation reveals normal and pathogenic THAP11 functions in gene expression and cell proliferation
Source: PLoS One. 2020 Jan 6;15(1):e0224646. doi: 10.1371/journal.pone.0224646 (PMC6944463; doi:10.1371/journal.pone.0224646)
Supplement: S8 Table — Green plus, interaction; red minus, no interaction; (—), very weak, if any, interaction; grey dot, not tested. See [40]. (PDF) [file pone.0224646.s017.pdf]

	THAP4	THAP5	THAP7	THAP8	THAP11
THAP7	•	•	+		
THAP8	•	•	•	+	
THAP11	—	•	—	+	+
HCF-1	—	(—)	+	+	+
